# Findings on Age at Onset of Cancer in Diabetic and Non-diabetic Populations

**DOI:** 10.7759/cureus.65719

**Published:** 2024-07-30

**Authors:** Ángel Gómez-Villanueva, Sharon I Martínez-Gómez, David E González-Mendoza, Edgar A Ramos-Gutiérrez, Roosvelth G Hernández-Ramírez, Lesly D Delgado-Villarejo, José J Garduño-García

**Affiliations:** 1 Oncology, Hospital General Regional No. 251, Instituto Mexicano del Seguro Social (IMSS), Metepec, MEX; 2 Internal Medicine, Hospital General de Zona No. 194, Instituto Mexicano del Seguro Social (IMSS), Naucalpan, MEX; 3 Medicine, Universidad Autónoma del Estado de México (UAEMex), Toluca, MEX; 4 Geriatrics, Hospital General Regional No. 251, Instituto Mexicano del Seguro Social (IMSS), Metepec, MEX; 5 Internal Medicine, Hospital General Regional No. 251, Instituto Mexicano del Seguro Social (IMSS), Metepec, MEX

**Keywords:** diagnosis, age at onset, metformin, diabetes, cancer

## Abstract

Background

Diabetes mellitus and cancer are two associated chronic diseases. Despite being a widely researched topic, the underlying mechanisms of this association remain unclear. One of the poorly explored topics regarding diabetes and cancer is the relation between the age of cancer onset and diabetes mellitus status; therefore, this research exposes the difference in the age of cancer diagnosis in both groups.

Methods

We conducted a retrospective study by reviewing the clinical files on a secondary care hospital's database. Files from first-time consultations of patients over 18 diagnosed using a histopathological report were included. The present study aimed to determine whether there is a difference in age at the onset of cancer in diabetic and non-diabetic individuals. Moreover, we calculated the average BMI at the onset for both populations.

Results

Our study included 8,741 patients; 1,551 (17.8%) were diabetic, and 7,190 (82.2%) were non-diabetic. From 28 types of cancer, 27 showed a difference in the age at the onset of cancer when diabetic and non-diabetic subjects were compared. This difference is significant as it suggests a potential link between diabetes and cancer, which could have implications for early detection and prevention strategies. Out of the 27 types, 17 showed statistical significance with p-values ranging from 0.048 to <0.0001 considering a 95% CI. Among those, the most significant types of cancer were breast, cervical, lung, ovarian, rectal, thyroid, and sarcoma, reporting p-values <0.0001. The mean age at onset of cancer in diabetic and non-diabetic populations was 62.7 years (SD ± 3.9) and 55.3 years (SD ± 7.9), respectively, showing a difference of 7.4 years in both groups. The BMI was statistically significant in patients with breast (p = 0.006), endometrial (p = 0.007), head and neck (p=0.014), and thyroid (p = 0.022) cancer types.

Conclusion

The data offer a critical view of the relationship between cancer and diabetes. Since virtually no one has produced a similar report, there is a broad field for researching the causal factors implicated in the pathway of diabetic and non-diabetic individuals who develop cancer. Research regarding metformin, diabetic neuropathy, and other possible causes must be addressed to determine whether they are involved in this process.

## Introduction

Type 1 and 2 diabetes mellitus are diseases related to an increased risk of cancer [1], among which one finds pancreatic, breast, cholangiocarcinoma, hepatocellular, colorectal, endometrial, gallbladder, and kidney cancers [2,3].

People affected by cancer and diabetes experience a torpid evolution. Their recurrence and mortality rates are by far more significant than those of individuals who only have diabetes or cancer, in addition to having a survival rate five years shorter and a considerable risk of hospitalization and infection development [4,5]. In Mexico, as noticed in 2022 official figures, the second and third causes of mortality were diabetes and cancer, with diabetes responsible for 115,681 deaths and cancer for 90,018 [6]. Based on statistics, the percentage of individuals with cancer and diabetes dropped between 8% and 18% for the entire cancer population [7,8].

Although the mechanisms that correlate cancer and diabetes are unclear, some scientists have illustrated possible pathways in their relationship. Researchers have mentioned that the association between diabetes and cancer is due to the mitogenic effects of insulin and insulin-like growth factor 1 (IGF-1) [9]. Others have established that malignant cells change their metabolism from oxidative phosphorylation into a glycolytic mechanism to obtain more energy, which helps the proliferation of cells. Furthermore, the bloodstream’s hyperglycemic state enhances this process [10].

Aging and hormones are two determinants of chronic diseases that are relevant in their development. In obese postmenopausal women with endometrial cancer, estrogens and hyperinsulinemia favor the growth of fatty tissue, creating a “vicious circle.” This circle produces a hyperinsulinemia state, increases hormones in the bloodstream (estrogen), and heightens cancer evolution [11,12]. Elevated BMI is another risk factor associated with cancer since it is linked to hyperinsulinemia, hyperglycemia, chronic inflammation, increased fatty tissue, and changes in hormonal balance [13]. Moreover, the persistence of fatty tissue in people with high BMI values furthers the production of proinflammatory molecular markers such as interleukin-6 (IL-6), adiponectin, leptin, and tumoral necrosis factor-alpha (TNF-α), which makes tumoral cells evolve into malignant ones [13,14].

As cancer and diabetes continue to pose significant public health challenges, research on potential treatments is a priority. In Mexico, metformin, a first-line anti-diabetes drug, is emerging as a promising candidate with not only hypoglycemic but also anti-cancer effects. This process involves activating AMPK proteins, modifying mitochondrial metabolisms, and reducing proinflammatory proteins such as IL-2, TNF-α, and interferon-gamma (INF-γ).

To our knowledge, there is only one paper that offers data on the existence of a difference in the age at cancer diagnosis in diabetic and non-diabetic individuals [15]; in this way, the present research showcases a difference in the age at cancer diagnosis in both groups.

## Materials and methods

Study design and participants

We performed a study by reviewing the clinical files on the database of the Regional General Hospital 251, a secondary care hospital in in Metepec, Mexico. As inclusion criteria, we considered first-time consultation patients older than 18 diagnosed using a histopathological report of any of the 28 types of cancer listed in the collection of data and variables. Biopsies that reported benign and hematological neoplasms were considered exclusion criteria. We included information from 8,741 participants who met the inclusion criteria and attended medical consultations from January 2011 to December 2023, while patients with incomplete or mistaken data, including height, weight, sex, sort of cancer, and histopathological biopsy reports, were excluded. We searched for information in PubMed, Google Scholar, and a web database. We utilized the EndNote 21.3 for macOS® (Clarivate, London, UK) for referencing.

Collection of data and variables

This study has as a goal to ascertain whether there is a difference in the age at the onset of cancer in diabetic and non-diabetic individuals, in addition to defining the average BMI at onset for both populations. Data were obtained from the individuals’ electronic clinical files, which included sex, height, weight, smoking and drinking habits, sort of cancer, histopathological biopsy report, age at onset, stage of cancer, date of diagnosis, and the presence or absence of diabetes at the time of such diagnosis. The BMI was obtained by dividing the patient’s weight (in kilograms) by the square of the height (in meters). Twenty-eight types of cancer were taken from the electronic clinical files of the Mexican Social Security Institute database, including head and neck, cervical, colorectal, endometrial, esophageal, gastric, gastrointestinal stromal tumor (GIST), gliomas, hepatocarcinoma, breast, malignant melanoma, mesothelioma, neuroendocrine, osteosarcoma, ovarian, pancreatic, penile, unknown primary cancer, prostate, lung, rectal, kidney, sarcoma, testicular, thyroid, gallbladder, cholangiocarcinoma, and non-classified cancer. The collected data were grouped for later analyses in contingency tables, comparing quantifiable data such as age and BMI and deleting files with missing data. Moreover, we compared the average age at the onset of cancer in diabetic and non-diabetic individuals and the average BMI of both populations. At the moment of selecting the sample for the analysis of the cancer stage at the initial diagnosis for both populations, 463 individuals were not considered because of absent data related to the cancer stage. 

Statistical analysis 

Initially, we executed the Shapiro-Wilk test to classify data regarding average BMI in subjects with cancer and diabetic or non-diabetic status. The average age at the onset of cancer in diabetic and non-diabetic individuals was categorized into parametric and non-parametric data; subsequently, we ran the Student's t-test and Mann-Whitney (MW) tests to identify whether there was a statistical significance (considered as a p-value ≤ 0.05) in parametric and non-parametric data, respectively, depending on their distribution. The statistical analysis was performed using Epi Info 7.2.2.6 (CDC, Atlanta, GA) and XLSTAT 2023.3.1 (1416) software (Lumivero, Denver, CO).

Ethical aspects

The Committee of Ethics Research 1503, attached to the Mexican Social Security Institute, rigorously reviewed and approved our study protocol (authorization code: F-2021-1503-082). This ethical approval and our commitment to international ethics guidelines, including the production and signing of a confidential agreement and dispensation letter, underscore our commitment to conducting this research with the utmost integrity and respect for patient privacy.

## Results

General study description

The sample comprised 8,741 participants; 1,551 (17.8%) were diabetic, and 7,190 (82.2%) were non-diabetic. The average age at onset for the diabetic population was 62.7 (SD ± 3.9) years, while for the non-diabetic, it was 55.3 (SD ± 7.9) years. The most frequent cancer type in both participant populations was breast cancer, with 476 (5.4%) diabetic and 2,164 (24.7%) non-diabetic individuals, equivalent to one-third of the sample. Table 1 contains characteristics and demographic population data. 

**Table 1:** Characteristics of the study population stratified by gender

Characteristics	Overall	Diabetic individuals diagnosed with cancer	Non-diabetic individuals diagnosed with cancer
Age, mean years ± SD	-	62.7 ± 3.9	55.3 ± 7.9
Sex, n (%)	-	-	-
Women	5900 (67.50)	1049 (67.63)	4851 (67.47)
Men	2841 (32.50)	502 (32.37)	2339 (32.53)
BMI (kg/m^2^), mean ± SD	-	25.8 ± 1.5	25.2 ± 1.4
Smoking habit, n (%)	-	-	-
Less than 1 packet per month	840 (9.61)	120 (7.74)	720 (10.01)
1-2 packets per month	581 (6.65)	86 (5.54)	495 (6.88)
2-3 packets per month	429 (4.91)	75 (4.84)	354 (4.92)
4+ packets per month	753 (8.61)	189 (12.19)	564 (7.84)
No consumption	6138 (70.22)	1081 (69.70)	5057 (70.33)
Drinking habit, n (%)	-	-	-
Social consumption	1678 (19.20)	292 (18.83)	1386 (19.28)
Once a week	495 (5.66)	80 (5.16)	415 (5.77)
Once a month	252 (2.88)	36 (2.32)	216 (3.00)
Once a year	187 (2.14)	34 (2.19)	153 (2.13)
No consumption	6129 (70.12)	1109 (71.50)	5020 (69.82)
Cancer stage at diagnosis, n (%)	8122	1444	6678
I	1055 (12.99)	174 (12.05)	881 (13.19)
II	1482 (18.25)	276 (19.11)	1206 (18.06)
III	1691 (20.82)	275 (19.04)	1416 (21.20)
IV	1886 (23.22)	377 (26.11)	1509 (22.60)
No stage available	2008 (24.72)	342 (23.68)	1666 (24.95)

Based on individuals’ sex (MW), the proportions between non-diabetics and diabetics were broadly similar in seven types of cancer, including breast cancer (4.3/4.5), cholangiocarcinoma (1.3/1.3), gastric cancer (3.0/2.6), hepatocarcinoma (1.3/1.4), mesothelioma (4.0/4.0), non-classified cancer (1.1/1.6), and pancreatic cancer (2.4/2.3). Meanwhile, the proportions between non-diabetics and diabetics were different in three types of cancer (MW), including esophageal cancer (7.3/3.7), glioma (8.2/2.3), and osteosarcoma (3.7/11.0). Only those with mesothelioma showed the same proportion (1.0) between both sexes, considering the diabetic and non-diabetic states.

The difference in age at cancer diagnosis between diabetic and non-diabetic populations was 7.4 years. The age at the onset of cancer was found to be statistically significant in 17 types of cancer, with a threshold of p <0.05. These include breast cancer with a difference of eight years (p <0.0001), cervical cancer with 6.8 years (p <0.0001), colon cancer with 6.5 years (p = 0.001), gastric cancer with 4.4 years (p = 0.034), GIST with 8.5 years (p = 0.029), head and neck cancer with 6.1 years (p = 0.03), hepatocarcinoma with 8.5 years (p = 0.048), kidney cancer with 4.8 years (p = 0.001), lung cancer with 7.2 years (p <0.0001), malignant melanoma with 8.9 years (p = 0.002), neuroendocrine cancer with 13.2 years (p = 0.037), non-classified cancer with 7.5 years (p = 0.003), ovarian cancer with 8.5 years (p <0.0001), rectal cancer with seven years (p <0.0002), sarcoma with 10.7 years (p <0.0001), testicular cancer with 25.4 (p <0.0001), and thyroid cancer with 13.4 years (p <0.0001). Even though only 17 exhibited statistical significance, in 27 out of 28 types of cancer, the diabetic population was diagnosed years later than the non-diabetic population. The complete information is shown in Table 2.

**Table 2:** Analysis of the average age of diabetic and non-diabetic populations at cancer diagnosis GIST: gastrointestinal stromal tumor; UPC: unknown primary cancer; MW: Mann-Whitney test; ST: Student's t-test *: p-values considered statistically significant

	Overall	Diabetic individuals diagnosed with cancer		Non-diabetic individuals diagnosed with cancer		
Primary cancer	Total cases, n (%)	Cases, n (%)	Average onset age	Cases, n (%)	Average onset age	p-value
Breast cancer	2640 (30.20)	476 (18.03)	61.9	2164 (81.97)	53.9	<0.0001* (MW)
Cervical cancer	570 (6.52)	96 (16.84)	58.7	474 (83.16)	51.9	<0.0001* (MW)
Cholangiocarcinoma	93 (1.06)	21 (22.58)	65.1	72 (77.42)	60.4	0.102 (ST)
Colon cancer	502 (5.74)	86 (17.13)	63.5	416 (82.87)	57.3	0.001* (MW)
Endometrial cancer	287 (3.28)	75 (26.13)	59.1	212 (73.87)	56.9	0.1 (MW)
Esophageal cancer	47 (0.54)	8 (17.02)	64.5	39 (82.98)	60	0.413 (MW)
Gallbladder cancer	135 (1.54)	27 (20.00)	63.8	108 (80.00)	63.1	0.779 (MW)
Gastric cancer	382 (4.37)	51 (13.35)	61.7	331 (86.65)	57.3	0.034* (ST)
GIST	93 (1.06)	15 (16.13)	65.6	78 (83.87)	57.1	0.029* (ST)
Glioma	91 (1.04)	9 (9.89)	56.1	82 (90.11)	47.1	0.11 (ST)
Head and neck	245 (2.80)	47 (19.18)	68	198 (80.82)	61.9	0.03* (MW)
Hepatocarcinoma	67 (0.77)	29 (43.28)	66.2	38 (56.72)	57.7	0.048* (MW)
Kidney cancer	337 (3.86)	90 (26.71)	62.3	247 (73.29)	57.5	0.001* (MW)
Lung cancer	220 (2.52)	43 (19.55)	68.8	177 (80.45)	61.2	<0.0001* (ST)
Malignant melanoma	185 (2.12)	33 (17.84)	67.5	152 (82.16)	58.6	0.002* (MW)
Mesothelioma	25 (0.29)	5 (20.00)	64.2	20 (80.00)	63.1	0.888 (MW)
Neuroendocrine cancer	68 (0.78)	8 (11.76)	63.1	60 (88.24)	49.9	0.037* (ST)
Non-classified cancer	265 (3.03)	56 (21.13)	67.8	209 (78.87)	60.3	0.003* (MW)
Osteosarcoma	26 (0.30)	4 (15.38)	54.5	22 (84.62)	37.3	0.087 (ST)
Ovarian cancer	501 (5.73)	69 (13.77)	58.1	432 (86.23)	49.6	<0.0001* (MW)
Pancreatic cancer	107 (1.22)	32 (29.91)	61.6	75 (70.09)	60	0.481 (ST)
Penile cancer	26 (0.30)	7 (26.92)	60.1	19 (73.08)	52.6	0.205 (ST)
Prostate cancer	458 (5.24)	97 (21.18)	70.3	361 (78.82)	70.2	0.787 (MW)
Rectal cancer	292 (3.44)	52 (17.81)	64.4	240 (82.19)	57.4	<0.0002* (MW)
Sarcoma	237 (2.71)	39 (16.46)	59.4	198 (83.54)	48.7	<0.0001* (ST)
Testicular cancer	471 (5.39)	9 (1.91)	56.6	462 (98.09)	31.2	<0.0001* (MW)
Thyroid cancer	225 (2.57)	34 (15.11)	61.3	191 (84.89)	47.9	<0.0001* (ST)
UPC	146 (1.67)	33 (22.60)	62.3	113 (77.40)	59.4	0.308 (MW)

The mean BMI was 25.8 kg/m^2^ (SD ± 1.5) for diabetic subjects and 25.2 kg/m^2^ (SD ± 1.4) for non-diabetic individuals. When analyzing Table 3, one notices that BMI at the onset of cancer in each population was statistically significant in four types: breast cancer with a difference of 0.7 (p = 0.006), endometrial cancer with a difference of 1.8 (p = 0.007), head and neck cancer with a difference of 1.6 (p = 0.014), and thyroid cancer with a difference of 2.1 (p = 0.022).

**Table 3:** Analysis of the BMI of diabetic and non-diabetic individuals diagnosed with cancer GIST: gastrointestinal stromal tumor; UPC: unknown primary cancer; MW: Mann-Whitney test; ST: Student's t-test *: p-values considered statistically significant

-	Overall	Diabetic individuals diagnosed with cancer		Non-diabetic individuals diagnosed with cancer		-
Primary cancer	Cases, n (%)	Cases, n (%)	BMI	Cases, n (%)	BMI	P-value
Breast cancer	2640 (30.20)	476 (18.03)	28	2164 (81.97)	27.3	0.006* (MW)
Cervical cancer	570 (6.52)	96 (16.84)	26.9	474 (83.16)	26.4	0.353 (MW)
Cholangiocarcinoma	93 (1.06)	21 (22.58)	25	72 (77.42)	25.5	0.876 (MW)
Colon cancer	502 (5.74)	86 (17.13)	24.4	416 (82.87)	23.8	0.237 (MW)
Endometrial cancer	287 (3.28)	75 (26.13)	29.8	212 (73.87)	28	0.007*(MW)
Esophageal cancer	47 (0.54)	8 (17.02)	23.1	39 (82.98)	22.7	0.79 (ST)
Gallbladder cancer	135 (1.54)	27 (20.00)	25.6	108 (80.00)	25.9	0.583 (MW)
Gastric cancer	382 (4.37)	51 (13.35)	24	331 (86.65)	23.1	0.16 (MW)
GIST	93 (1.06)	15 (16.13)	25.4	78 (83.87)	24.8	0.98 (MW)
Glioma	91 (1.04)	9 (9.89)	27.1	82 (90.11)	25.8	0.173 (MW)
Head and neck	245 (2.80)	47 (19.18)	27.2	198 (80.82)	25.6	0.014* (MW)
Hepatocarcinoma	67 (0.77)	29 (43.28)	24.8	38 (56.72)	25.6	0.579 (MW)
Kidney cancer	337 (3.86)	90 (26.71)	26.7	247 (73.29)	25.9	0.138 (MW)
Lung cancer	220 (2.52)	43 (19.55)	24.8	177 (80.45)	24.1	0.25 (MW)
Malignant melanoma	185 (2.12)	33 (17.84)	25.3	152 (82.16)	26.3	0.218 (ST)
Mesothelioma	25 (0.29)	5 (20.00)	23.8	20 (80.00)	23.5	0.878 (TS)
Neuroendocrine cancer	68 (0.78)	8 (11.76)	26.1	60 (88.24)	24.1	0.46 (MW)
Non-classified cancer	265 (3.03)	56 (21.13)	26.3	209 (78.87)	25.7	0.772 (MW)
Osteosarcoma	26 (0.30)	4 (15.38)	25.5	22 (84.62)	23.9	0.56 (ST)
Ovarian cancer	501 (5.73)	69 (13.77)	26.6	432 (86.23)	26	0.204 (MW)
Pancreatic cancer	107 (1.22)	32 (29.91)	23.5	75 (70.09)	22.8	0.212 (MW)
Penile cancer	26 (0.30)	7 (26.92)	25.2	19 (73.08)	28.3	0.171 (ST)
Prostate cancer	458 (5.24)	97 (21.18)	26.2	361 (78.82)	25.4	0.416 (MW)
Rectal cancer	292 (3.44)	52 (17.81)	24.6	240 (82.19)	23.9	0.645 (MW)
Sarcoma	237 (2.71)	39 (16.46)	26.2	198 (83.54)	25.3	0.26 (MW)
Testicular cancer	471 (5.39)	9 (1.91)	26.1	462 (98.09)	25	0.168 (MW)
Thyroid cancer	225 (2.57)	34 (15.11)	28.5	191 (84.89)	26.4	0.022* (MW)
UPC	146 (1.67)	33 (22.60)	25.2	113 (77.40)	24.5	0.422 (MW)

## Discussion

Our study not only unveiled data on the relationship between BMI, diabetes, and cancer but also unearthed novel data on the age at cancer onset in diabetic and non-diabetic individuals. The discovery in our study of the 7.4-year difference is a unique and significant finding, suggesting that non-diabetic patients may be at a higher risk of developing cancer at an earlier age than diabetic patients. This statistically significant age difference in most cancer types is a unique finding and could potentially lead to substantial advancements in our understanding of these diseases. While we cannot disclose the source of this finding, as our focus was on numerical data, we sought out studies that could shed light on these novel findings.

Many studies describe a link between cancer and diabetes; factors such as hyperglycemia, hyperinsulinemia, insulin resistance, obesity, oxidative stress, inflammation, increased leptin, and decreased adiponectin levels, increased IGF-1 concentrations, and genetics have been investigated as critical components in the relation between cancer and diabetes [14, 16, 17]. Nevertheless, only one study has mentioned the connection between diabetes and age at the onset of cancer. In this study, the authors found that diabetic subjects diagnosed with pancreatic cancer were older than the non-diabetic subjects (68.6 years at diagnosis and 60 years at diagnosis, respectively). For their part, diabetic individuals had an average BMI of 29.7 kg/m^2^, a higher BMI than those in the non-diabetic population, representing a BMI of 24.9 kg/m^2^ [15]. The results regarding age were similar to those of our study; even though only 17 types were statistically significant, they generally applied to most cancer types. In the case of BMI results, it differed a little because the vast majority of our population was obese or overweight. If we increased the sampled population, the data would probably behave similarly to that reported in the study above.

We searched for other papers that might explain why diabetic people are diagnosed with cancer at older ages than those deemed "healthy." We could not find accurate information, though we considered two possible hypotheses. The first is based on the use of antidiabetic drugs by diabetic individuals, such as metformin, which has been evaluated to have an antineoplastic effect, delaying the onset of cancer [1, 15]. The second, which, to our knowledge, has not been documented, could have a basis in the presence of diabetic neuropathy, a status that affects the nerves and the perception of symptoms. We believe these mechanisms could delay cancer diagnosis; however, they are only hypotheses, and more research is needed to prove them. It is important to note that our current research has limitations, and further studies are necessary to validate our findings. 

Today, the widespread use of antidiabetic drugs, driven by the high prevalence of metabolic syndrome, obesity, and diabetes, has opened up new avenues for research. This has led to the discovery of the potential anticarcinogenic effect of certain antidiabetic drugs, particularly metformin. We hypothesized that metformin could influence the progression of cancer types with a later onset in diabetic subjects. The majority of investigations into the relationship between metformin and delayed cancer onset have shown a reduction in cancer incidence and mortality in diabetic subjects taking this drug, offering a glimmer of hope in the fight against cancer [18-20].

Metformin is an antineoplastic drug that inhibits tumorigenesis by acting at cellular (direct effects) and systemic (indirect effects) levels [21,22]. At the cellular level, metformin penetrates the mitochondria. It modifies the respiratory complex I in oxidative phosphorylation, which leads to lower production of adenosine triphosphate (ATP), a vital energetic molecule that normal and cancer cells use for their metabolism [23]. At the systemic level, it suppresses gluconeogenesis and glycogenolysis, which results in lower serum glucose levels, decreased gastrointestinal absorption of glucose, and reduced circulation of insulin; this hormone can promote tumorigenesis [22]. Such reduction in ATP molecules activates AMPK, an enzyme critical as an energy sensor and tumor suppressor, inhibiting cell proliferation and saving energy via enhancing glucose uptake and glycolysis [23]. Other mechanisms that have been related to metformin as an anti-cancer drug are the activation of factors that inhibit the cell cycle (p53, p21, cyclin D1) as well as cell proliferation, which induces apoptosis [22,24], the suppression of hypoxia-inducible factor (HIF)-1 and vascular endothelial growth factor (VEGF), making cancer cells more susceptible to hypoxic environments, metastasis, and wrong angiogenesis development [25].

Some other studies researched metformin’s association with a reduced risk of colon cancer, demonstrating an increased survival rate among those who were medicated with the drug [26-29]. In a meta-analysis by Mei and colleagues, the examination of 21 observational studies on metformin and colon cancer showed a reduction in mortality with a hazard ratio of 0.65 (95% CI 0.56-0.76) [30]. 

We faced some limitations when looking for information to support the hypothesis that puts forward the existence of relevance in the difference in the age of cancer diagnosis in diabetic and non-diabetic subjects. As only one study has reported data similar to ours, there is a critical field to explore this novel discovery in Mexican populations and other populations with different genetic compositions and lifestyles to test if there is a similarity or a difference. In that way, we could not conclude if it was just an incidental finding proper to our population or if this follows a particular pattern regardless of how different the populations are as long as they are diagnosed with diabetes and cancer.

## Conclusions

The data in this study offer a glimpse into the way our population is affected by cancer; it will be crucial for local authorities to understand the size of the oncological population and make decisions regarding medications and management. This information may also motivate similar hospitals to investigate their local oncological statistics. While there is evidence of the relation between cancer and diabetes, no reported findings are equivalent to those in the present study. There is much to explore about the association between the age at the onset of cancer in diabetic and non-diabetic individuals to identify if there is an actual relationship, not only for pancreatic cancer but also for other cancers. One key factor in this discovery may be medication with metformin, a drug widely used by our diabetic population. Moreover, further research on our population is necessary to either confirm or reject this assertion.
